# Inhibition of Hedgehog Delays Liver Regeneration through Disrupting the Cell Cycle

**DOI:** 10.3390/cimb44020032

**Published:** 2022-01-18

**Authors:** Jiawang Tao, Yan Chen, Yuanqi Zhuang, Ruzhi Wei, Anteneh Getachew, Tingcai Pan, Fan Yang, Yinxiong Li

**Affiliations:** 1 Institute of Public Health, Guangzhou Institutes of Biomedicine and Health (GIBH), Chinese Academy of Sciences (CAS), Guangzhou 510530, China; tao_jiawang@gibh.ac.cn (J.T.); chen_yan@gibh.ac.cn (Y.C.); zhuang_yuanqi@gibh.ac.cn (Y.Z.); wei_ruzhi@gibh.ac.cn (R.W.); anteneh@gibh.ac.cn (A.G.); pan_tingcai@gibh.ac.cn (T.P.); 2 University of Chinese Academy of Sciences, Beijing 100049, China; 3 Ministry of Education CNS Regeneration Collaborative Joint Laboratory, Guangdong-Hongkong-Macau Institute of CNS Regeneration, Jinan University, Guangzhou 510632, China; yang_fan@gibh.ac.cn; 4 Guangdong Provincial Key Laboratory of Stem Cell and Regenerative Medicine, Guangzhou 510530, China; 5 Bioland Laboratory (Guangzhou Regenerative Medicine and Health Guangdong Laboratory), Guangzhou 510005, China; 6 State Key Laboratory of Respiratory Disease, Guangzhou 510530, China

**Keywords:** vismodegib, smoothened, liver regeneration, hedgehog signaling, mitosis

## Abstract

Liver regeneration is a complicated biological process orchestrated by various liver resident cells. Hepatic cell proliferation and reconstruction of the hepatic architecture involve multiple signaling pathways. It has been reported that the Hh signal is involved in liver regeneration. However, the signal transduction pathways and cell types involved are ill studied. This study aimed to investigate hedgehog signal response cell types and the specific molecular mechanism involved in the process of liver regeneration. Partial hepatectomy (PH) of 70% was performed on ICR (Institute of Cancer Research) mice to study the process of liver regeneration. We found that the hedgehog signal was activated significantly after PH, including hedgehog ligands, receptors and intracellular signaling molecules. Ligand signals were mainly expressed in bile duct cells and non-parenchymal hepatic cells, while receptors were expressed in hepatocytes and some non-parenchymal cells. Inhibition of the hedgehog signal treated with vismodegib reduced the liver regeneration rate after partial hepatectomy, including inhibition of hepatic cell proliferation by decreasing Cyclin D expression and disturbing the cell cycle through the accumulation of Cyclin B. The current study reveals the important role of the hedgehog signal and its participation in the regulation of hepatic cell proliferation and the cell cycle during liver regeneration. It provides new insight into the recovery of the liver after liver resection.

## 1. Introduction

The liver is a special organ with a strong ability to regenerate. After partial hepatectomy (PH), the liver can recover its original mass within two weeks [1]. A large number of cytokines and signaling pathways function in this process, including the hepatocyte growth factor (HGF), epidermal growth factor (EGF), transforming growth factor-β (TGFβ) and the Wnt and Hh signal pathways [1,2,3,4]. Furthermore, liver regeneration is a very complicated process that requires stages of initiation, proliferation and termination. After PH, the liver rapidly changes and displays characteristics of portal venous pressure, increased expression of β-catenin and HGF and upregulation of other cytokines [5,6,7]. Many studies have shown that cell proliferation during liver regeneration peaks at about 48 h post-PH, which involves the replication of mature hepatocytes [2,8]. In some liver injury models such as chronic liver injury, hepatectomy and drug toxicity models, liver progenitor cells may be the main cell types contributing to liver regeneration, serving as the second defensive line of the liver [9]. Recent studies have indicated that hedgehog (Hh) signaling plays an important role in many types of chronic liver injury, and even in hepatocellular carcinoma [10,11,12,13,14]. In the process of liver fibrosis, Hh signaling could be activated in hepatic stellate cells (HSCs), promoting further activation and proliferation of HSCs, leading to fibrogenesis [15,16,17]. In some studies, HSCs were considered as epithelial progenitors in the liver [18].

Hh is a developmental morphogenic signal with two transmembrane receptors, Patched (PTC) and Smoothened (SMO). When Hh ligands bind to PTC, PTC relieves its inhibitory effect on SMO. SMO is a signaling transducer that causes the downstream uncoupling of the negative regulator Su(fu) protein from glioma-associated oncogene (GLI) transcription factors. The subsequent nuclear translocation and DNA binding of GLI1, GLI2 and possibly GLI3 are followed by the increased transcription of a number of genes, including PTC itself [19,20,21]. Many studies have demonstrated that Hh signaling is critical for the cell proliferation and differentiation of stem cells, progenitor cells and tumor cells [22,23,24]. Some studies indicated that Cyclin D was suppressed in SMO knock-out cells [10]. It has been demonstrated that Cyclin D was the downstream gene of the Hh signaling pathway [25,26]. In addition, PTC could interact with the phosphorylation of Cyclin B1 and then alter the localization of the maturation-promoting factor (MPF), which regulates cell cycle progression [27,28,29]. Cyclin B, as a mitotic cyclin, functions throughout mitosis. Successfully complete mitosis of the cell needs the degradation of Cyclin B regulated by the anaphase-promoting complex (APC/C) [30,31]. APC/C is a significant complex regulating cell division, of which the pivotal component Cdc20 is regulated by multiple checkpoint proteins, including MAD2, BUB3 and BUB1B [32,33]. However, the relationship between checkpoint proteins and Hh signaling remains poorly understood. Moreover, the more detailed roles of Hh signaling in cell proliferation and mitosis still require verification.

Since Hh signaling is involved in the cell cycle, it is worth asking whether it participates in liver regeneration. One study indicated that Hh is critical for normal liver regeneration. In a PH mouse model, treatment with the Hh inhibitor cyclopamine after 2/3 PH caused all the mice to die within 48 h [34], making it difficult to understand the mechanism of Hh in the inhibition of liver regeneration.

Vismodegib, which is structurally unrelated to cyclopamine, was the first Hh pathway inhibitor approved by the FDA (Food and Drug Administration) to be used in the treatment of basal cell carcinoma [35,36]. It inhibits Hh signaling by binding to SMO with high affinity and specificity. It also proved to be efficient in reducing TRAIL-mediated nonalcoholic liver injury [37] and attenuating liver fibrosis in bile duct ligated rats [38]. In addition, compared to the *Gli*-luc IC_50_ of cyclopamine of 500 nM, vismodegib had a much lower IC_50_ of 3 nM [39], which revealed the efficacy and safety of vismodegib. Our results show that SMO inhibition by vismodegib significantly decreased the liver regeneration rate without causing the death of mice. This reveals the important role of the Hh signal in liver regeneration after PH.

## 2. Materials and Methods

### 2.1. Animal Experiment

ICR male mice were used as a liver regeneration model, which were obtained from and maintained at the GIBH (Guangzhou Institutes of Biomedicine and Health) (Guangzhou, China) animal experimental center. Animal welfare and operation experiments were in accordance with the rules and regulations.

The PH protocol was based on that of Higgins and Anderson, with modifications [40,41]. The experimental group (9−11-week-old ICR male mice) was intragastrically administered vismodegib (20 mg/kg/day) (Selleck, Shanghai, China) for 3 days before PH and treated daily until sacrifice; sodium carboxymethylcellulose (CMC-NA) (Aladdin, Shanghai, China) was administered intragastrically to the control group. The mice were sacrificed at 1 day (*n* = 5), 2 days (*n* = 5), 3 days (*n* = 5), 7 days (*n* = 5) and 10 days (*n* = 5) after PH. Body weight was weighed every day until sacrifice. The resectioned part of the liver and the rest of the liver were weighed after PH and sacrifice, respectively. To study the rate of cell proliferation between the control and vismodegib groups, Edu (RiboBio, Guangzhou, China) was administered intraperitoneally (50 mg/kg weight) 2 h before sacrifice 36 h and 44 h after PH.

### 2.2. Real-time Quantitative PCR

Total RNA was isolated from liver tissue stored in liquid nitrogen by Trizol. Real-time PCR was performed with Bio-Rad CFX96 (BIO-RAD, Hercule, CA, USA). The experiment procedure was carried out according to the reagent instructions. Relative mRNA expression was analyzed by normalization with GAPDH (IGE, Guangzhou, China) in all genes. The sequence of primers is summarized in Supplementary Table S1.

### 2.3. Western Blot Analysis

Total protein was isolated from liver tissue by the RIPA (Radio Immunoprecipitation Assay) lysate (Beyotime, Guangzhou, China). Protein bands were separated by 10% SDS-PEGA (sodium dodecyl sulfate polyacrylamide gel electrophoresis) (Beyotime, Guangzhou, China) and transferred by the PVDF (polyvinylidene fluoride) membrane (Roche, Shanghai). Using 5% defatted milk (Beyotime, Guangzhou, China) blocked nonspecific binding, and the primary antibody was incubated at 4 °C overnight. Antibodies against GLI1 and GLI2 were purchased from GeneTex; Cyclin D and Cyclin B from Abcam; and Bub1b, Cdc20 and CDK2 from Poteintech.

### 2.4. Immunohistochemistry

Liver tissues were fixed with 4% paraformaldehyde (PFA) (Beyotime, Guangzhou) overnight and then cut into slices through paraffin embedding. Slice staining was followed by routine staining procedures with deparaffinization in xylene (Guangzhou Chemical Reagent Factory, Guangzhou, China), antigen unmasking in boiling sodium citrate buffer (Beyotime, Guangzhou, China) and incubation in 3% hydrogen peroxide (HARVEYBIO, Beijing, China) for fewer false positives and a clear background. The antibodies used were the same as those for the Western blot.

### 2.5. Isolation of Primary Hepatocytes and Flow Cytometry Analysis

Primary hepatocytes were isolated from normal and PHx mice through two-step collagenase digestion and differential centrifugation. Polyploid cells were analyzed using Hoechst 33342 staining (Beyotime, Guangzhou, China). Samples were measured on LSR Fortessa SORP and analyzed with Flow Jo.10 (https://www.flowjo.com/, accessed on 2 January 2022).

Statistical analysis—Statistical significance was determined with an unpaired, two-sided Student *t* test using GraphPad Prism 6 (https://www.graphpad.com/scientific-software/prism/, accessed on 2 January 2022). Data were presented as means ± SEM, and standard errors were considered statistically significant at * *p* ≤ 0.05, ** *p* ≤ 0.01 and *** *p* ≤ 0.001.

## 3. Results

### 3.1. Hedgehog Signal Activated after Partial Hepatectomy

**Figure 1:**
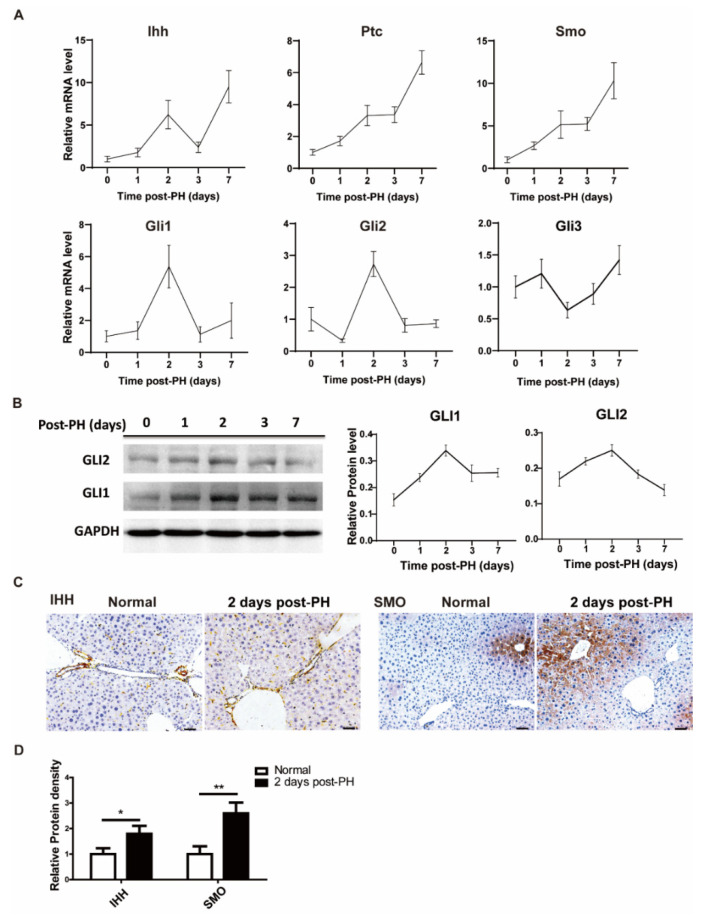
**Hedgehog signal activated after PH.** (**A**) mRNA change trend of Hh signaling pathway (*IHH*, *PTC*, *SMO*, *GLI1*, *GLI2*, *GLI3*) in liver tissue of mice at different time points after PH (0, 1, 2, 3 and 7 days), *n* = 5. (**B**) Representative Western blot results of GLI1 and GLI2 in the liver tissues of mice at different time points after PH (0, 1, 2, 3 and 7 days). Western blot quantification analysis compared to GAPDH is shown in the line chart, *n* = 3. (**C**) Representative immunohistochemical results of IHH and SMO staining in liver of normal mice and two days after PH, bar means 50 µm. (**D**) Results of IHH and SMO immunohistochemical quantitative analysis, analyzed with Image J, 5 pictures for each group, *n* = 3. All data represent means ± SEM. * *p* < 0.05, ** *p* < 0.01 vs. control group.

To investigate the changes in the Hh signal in the process of liver regeneration, 70% partial hepatectomy was performed on ICR mice, and liver tissue samples were collected at different time points (1, 2, 3 and 7 days). We first analyzed Hh signal-related expressions at the protein and mRNA levels. The results show that overall signal molecules were upregulated after PH at the mRNA level. Among them, the receptors (*PTC* and *SMO* were continuously increased during regeneration, and the ligand (*IHH*) had two high-expression points (2 days and 7 days after PH). Likewise, downstream activation signals such as *GLI1* and *GLI2* were significantly upregulated at 2 days after PH, while the downstream inhibition signal *GLI3* was relatively downregulated 2 days after PH (Figure 1A). Correspondingly, GLI1 and GLI2 had similar trends in their mRNA and protein levels (Figure 1B). We next sought to determine specific cells expressing the Hh signal before and after PH. For this purpose, we stained tissue sections of normal and PH-induced mice using immunohistochemistry analysis. The results show that the Hh ligand, IHH, was mainly expressed in hepatic non-parenchymal cells (including endothelial cells and hepatic stellate cells) and bile duct cells of PH-induced mice; however, a mild expression was also observed in normal control mice. These results are consistent with the results obtained from mRNA analysis. On the other side, we observed a significant expression of the Hh receptor SMO in hepatocytes in addition to hepatic non-parenchymal cells, as shown in Figure 1C. Overall, this spatiotemporal appearance and synchronization of Hh signal molecules and receptors may imply that Hh signal molecules were secreted from hepatic non-parenchymal cells and bile duct cells and acted on hepatocytes to promote liver regeneration.

### 3.2. Vismodegib Suppresses the Rate of Liver Regeneration after PH

**Figure 2:**
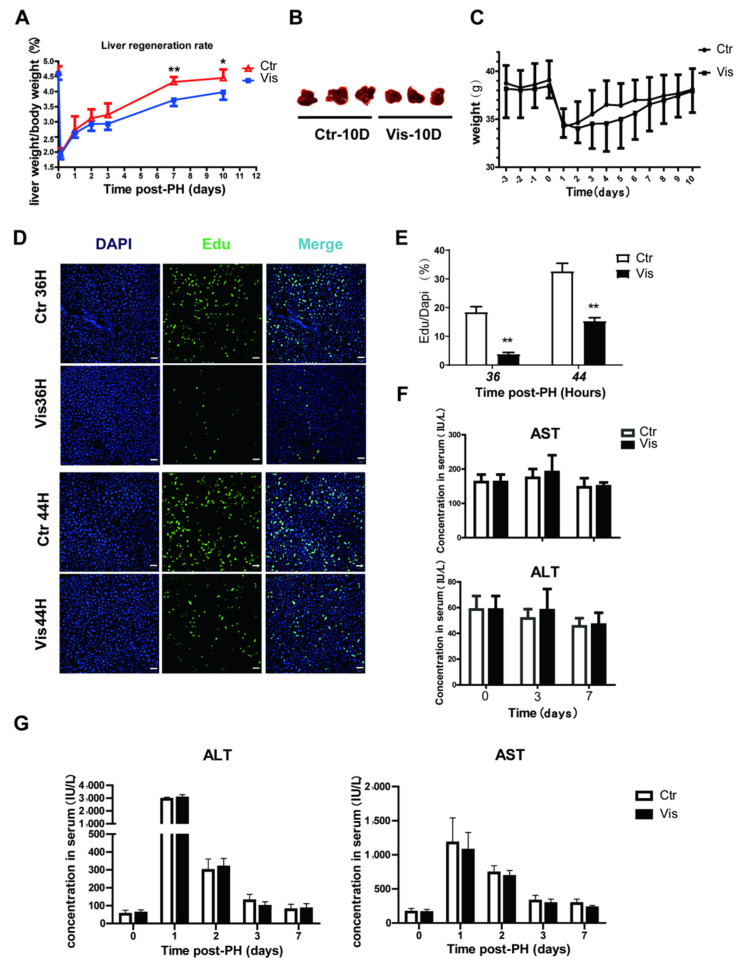
**Vismodegib suppresses the rate of liver regeneration after PH.** (**A**) Liver-to-body weight ratios of control and vismodegib-treated mice at different times after 70% partial hepatectomy (0, 3 h, 1 day, 2 days, 3 days, 7 days and 10 days), *n* = 5. (**B**) Images of mouse livers at 10 days post-PH between the two groups, *n* = 3. (**C**) The change in the body weight of the control and vismodegib-treated mice between 3 days pre-PH and 10 days post-PH; the trends of the two groups were consistent, *n* = 5. (**D**) Edu was used to analyze the difference in proliferative cell amounts between the normal and vismodegib-treated groups after PH. Representative immunofluorescence results 36 h and 44 h after PH. (**E**) Quantification percentage of Edu positive cells to DAPI positive cells shown in the histogram. (**F**) Changes in liver function index of normal mice treated with vismodegib (AST: aspartate aminotransferase, ALT: alanine aminotransferase) at different times (0, 3, 7 days). There was almost no difference between the two groups, *n* = 5. (**G**) Changes in liver function index (ALT and AST) of mice at different times post-PH in the two groups, *n* = 5. All data represent means ± SEM. * *p* < 0.05, ** *p* < 0.01 vs. control group.

In healthy mice, liver-to-body weight ratios are constant at around 4–5% [1,3]. Studies have demonstrated that in rodents, the liver has the ability to recover to normal mass within 10 days after PH [3,42]. In our study, the liver of control group mice recovered more than 90% of its normal weight at 10 days after PH. However, mice treated with vismodegib showed a lower liver mass, and liver regeneration was inhibited at all the collected time points after PH. Especially by 7 to 10 days after PH, the liver-to-body weight ratio had decreased by approximately 20% of the control group (Figure 2A). The images of mouse livers 10 days post-PH also showed a significantly decreased degree of regeneration in the vismodegib-treated group (Figure 2B), in addition to no significant difference in body weight between the two groups (Figure 2C). We further validated this result by using Edu (5-Ethynyl-2′-deoxyuridine) incorporation data. Edu is a nucleic acid analogue that inserts DNA during DNA replication [43,44]. We could measure the amount of cell proliferation by detecting Edu. DNA synthetic activities of hepatocytes reached a peak between 24 h and 48 h post-PH. We chose 36 h and 44 h as the time points for measuring DNA synthetic activity. The results indicate that incorporation of Edu decreased noticeably in the hepatic cells of vismodegib-treated mice compared with that in the control group. At 36 h post-PH, the Edu-labeled cell nuclei decreased by 80%, and then by almost 60% at 44 h post-PH, in the vismodegib-treated group (Figure 2D,E). Furthermore, the vismodegib-treated mice showed comparable survival rates to their control-treated counterparts, as shown in Figure 2B, and the change trends of weight between the two groups were almost the same. In order to examine whether the administered vismodegib caused hepatotoxicity and affected liver regeneration, the same doses of vismodegib were applied to normal mice in the same manner to the experimental groups, and the levels of ALT (alanine aminotransferase) and AST (aspartate transaminase) were measured on days 3 and 7. There was no significant difference between the control and vismodegib-treated groups (Figure 2F). Furthermore, ALT and AST did not differ significantly between the two groups after PH (Figure 2G). It was proved that 20 mg/kg vismodegib caused no hepatotoxicity effect in the mouse livers. The results show that inhibition of the Hh signal pathway with vismodegib significantly reduced the liver regeneration rate after PH.

### 3.3. Vismodegib Inhibits the Activation of Hh Pathway after PH

**Figure 3:**
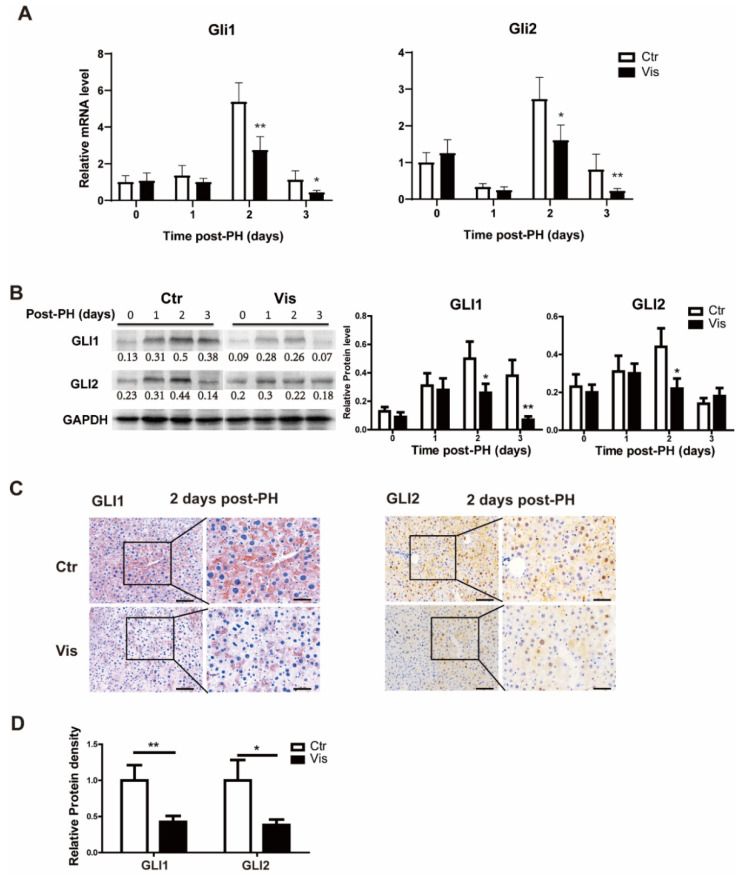
**Vismodegib inhibits the activation of the Hh pathway after PH.** (**A**) QRT-PCR analysis of Hh signaling pathway intracellular signaling molecules *GLI1* and *GLI2* in liver tissue at different times after PH (0, 1, 2 and 3 days), *n* = 5. (**B**) Representative Western blot analysis of GLI1 and GLI2 in the liver tissue between the two groups at different times after PH (0, 1, 2 and 3 days). Western blot quantification analysis compared to GAPDH is shown under the bands. (**C**) Representative immunohistochemical pictures of GLI1 and GLI2 in the liver slices of mice at two days after PH between the two groups. (**D**) Results of GLI1 and GLI2 immunohistochemical quantitative analysis of the two groups at 2 days post-PH are shown in the histogram, analyzed with Image J, 5 pictures for each group, *n* = 3. All data represent means ± SEM. * *p* < 0.05, ** *p* < 0.01 vs. control group.

As the most critical effector molecules of the Hh signal, GLI1 and GLI2 are involved in the transcriptional regulation of various genes. They are also regulated by the Hh signal. To determine changes in the Hh signal after the administration of vismodegib, GLI1 and GLI2 were detected at both the mRNA and protein levels in the mouse liver tissue at different time points after PH. There were no significant changes in GLI1 and GLI2 at the early stage of hepatectomy (about 1 day after PH) between the vismodegib-treated group and the control group. Meanwhile, at the peak of cell proliferation of liver regeneration (2–3 days after PH), GLI1 and GLI2 significantly decreased at both the mRNA and protein levels (Figure 3A–C) in the vismodegib-treated group. Immunohistochemistry analysis revealed that GLI1 and GLI2 expressions in both hepatocytes and non-parenchymal cells were consistent with SMO localization (Figure 3C). This indicated that hepatocytes, as the target cells of the Hh signal, were regulated by the Hh signal in the process of liver regeneration after PH. Taken together, vismodegib inhibited the Hh signaling pathway in hepatocytes, which was observed at the peak stage of cell proliferation, indicating that the Hh signal may be involved in the regulation of cell proliferation.

### 3.4. Inhibition of Hh Signal Suppressed the G1/S Phase of Cell Proliferation

**Figure 4:**
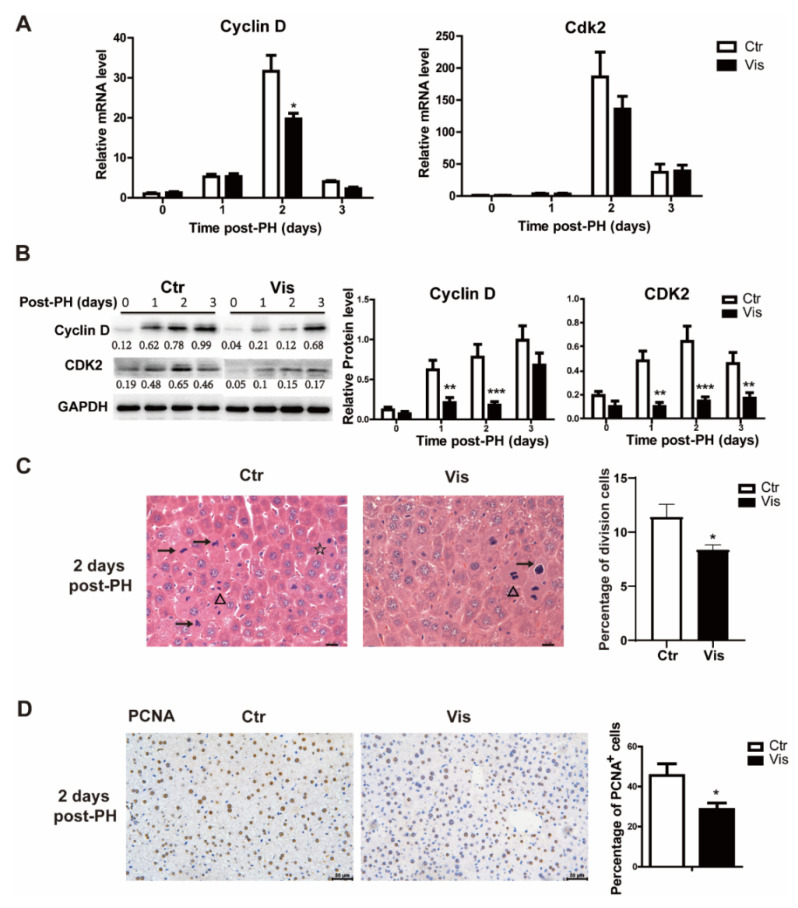
**Vismodegib inhibits cell proliferation in the cell cycle at the G1/S phase**. (**A**) QRT-PCR analysis of Cyclin D and Cdk2 in the liver tissue at different times after PH between the two groups (1, 2 and 3 days), *n* = 5. (**B**) Western blot analysis of Cyclin D and Cdk2 in liver tissue at different times after PH. Western blot quantification analysis compared to GAPDH is shown under the bands. (**C**) Quantification of the percentage of cells in different phases of cell division in H&E-stained slices. Arrow (→) represents cells of pre-division, triangle (Δ) means cells in division and star (☆) means cells of post-division. Division cells include the three mentioned above. Five full pictures of 20× were counted for each group. Percentage analysis compared to the total cells is shown in the histogram. (**D**) Representative immunohistochemical pictures of PCNA in the liver slices of mice at two days after PH between the two groups. Quantitative analysis is shown in the histogram, *n* = 3. All data represent means ± SEM. * *p* < 0.05, ** *p* < 0.01, *** *p* < 0.001 vs. control group.

During liver regeneration, the rest of the liver cells need to reenter the cell cycle from the G1 phase and begin cell proliferation to recover to the normal liver weight [1,3]. Cyclin D is an important cyclin protein involved in regulating cell cycle progression, which drives G1/S phase transition. In our study, mice treated with vismodegib had a lower expression of Cyclin D, and there was no significant inhibitory effect observed at the peak level of expression. In addition, the Cyclin D-related cyclin-dependent kinase *Cdk2* was also suppressed at 2 days post-PHx (Figure 4A). Western blot analysis also confirmed this result, as shown in Figure 4B. H&E staining of tissue sections of samples obtained from vismodegib-treated mice showed a limited number of dividing cells compared with the control groups, which demonstrated a lower rate of cell proliferation (Figure 4C). Furthermore, we detected PCNA (a marker gene for cell proliferation) in liver slices of the two groups. The results verify that there was less cell proliferation in vismodegib-treated mice compared with the control group (Figure 4D).

### 3.5. Inhibition of Hh Signaling Increased Accumulation of Polyploid Cells by Suppressing Degradation of Cyclin B

**Figure 5:**
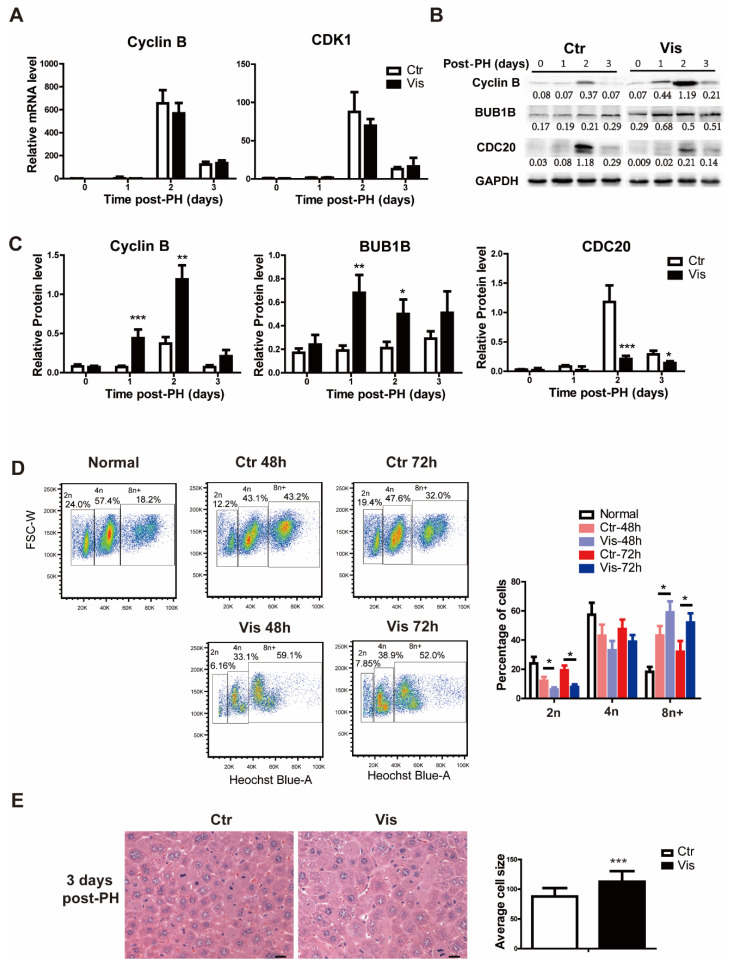
**Vismodegib increases the accumulation of polyploid cells through suppressing the degradation of Cyclin B.** (**A**) QRT-PCR analysis of *Cyclin B* and *Cdk1* in the liver tissue at different times after PH between the two groups (1, 2 and 3 days), *n* = 5. (**B**) Western blot analysis of Cyclin B, Cdc20 and Bub1b in liver tissue at different times after PH. Western blot quantification analysis compared to GAPDH is shown under the bands. (**C**) Quantification analysis of Cyclin B, CDC20 and BUB1B Western blot results is shown in the histogram. (**D**) Cell flow analysis of the proportion of polyploid primary hepatocytes by Hoechst 33342 in normal liver, and two groups of mice two days and three days after PH. The proportions of different polyploid cells are shown in the histogram. (**E**) H&E staining of liver slices at 3 days after PH showed different cell sizes between the two groups. A total of 100 cells were analyzed in each group. Cell sizes were analyzed by Image Pro Plus. Data represent means ± SEM. * *p* < 0.05, ** *p* < 0.01, *** *p* < 0.001 vs. control group.

In the process of the cell cycle, different cyclins play different roles at different stages. Cyclin B, as a mitotic cyclin binding to CDK1, forms the Cyclin B–CDK1 complex, which regulates the progression of cells into and out of the M phase. Degradation of Cyclin B is a prerequisite to allowing cell exit out of the mitosis phase. In our study, *Cyclin B* and *Cdk1* caused no significant change in the mRNA level in the vismodegib group (Figure 5A). However, at the protein level, the vismodegib group presented greater accumulation of Cyclin B (Figure 5B,C), which indicates the failure of cells to exit during mitosis. The results of cell sorting verify the hypothesis that inhibiting Hh signaling results in hepatocytes being trapped in the mitosis phase or lacking the ability to process mitosis and cytokinesis. In a healthy liver, normal hepatocytes are diploid, and mature hepatocytes are mostly polyploid. After PH, polyploid hepatocytes in the liver increased significantly to restore the liver to its normal size, as reported previously [45,46]. More surprisingly, the vismodegib group displayed a higher proportion of polyploid hepatocytes (Figure 5D). In addition, hematoxylin-eosin staining of liver sections obtained from the vismodegib-treated group detected significantly bigger cells as compared with the non-treated control groups (Figure 5E). Subsequently, the Western blot results showed that CDC20 participating in the ubiquitination and degradation of Cyclin B decreased in the vismodegib group, while the protein BUB1B inhibiting the degradation of Cyclin B was significantly increased (Figure 5B,C), corresponding to the accumulation of Cyclin B in the vismodegib group. In conclusion, inhibition of the Hh signal disrupted the cell cycle of hepatocytes, resulting in more polyploid cells and thereby inhibiting liver regeneration through inhibiting the degradation of Cyclin B.

## 4. Discussion

Liver regeneration following PH is a highly complex process involving harmony between a variety of cells, including hepatic parenchymal cells and non-parenchymal cells, in addition to cytokines promoting cell proliferation [2]. In recent decades, many researchers have demonstrated how these cells are involved in regeneration; however, the signaling pathways have not been fully discovered yet. Hh signaling is one of the pathways that mediate cell regeneration. Studies have demonstrated that Hh signaling is activated immediately after the induction of PH in the mouse liver. This could be manifested in the increasing expression of Hh ligands (IHH, SHH), receptors (SMO, PTC) and intracellular signals (GLI1, GLI2). Furthermore, the SMO inhibitor cyclopamine significantly inhibited mouse liver regeneration, even causing the death of mice after PH [34]. In another liver regeneration model with the novel two-staged hepatectomy (ALPPS), the Hh signaling pathway regulated the early acceleration of liver regeneration [47]. However, the detailed mechanism between Hh signaling and liver regeneration remains unclear. Our present study demonstrates that Hh signaling mediates liver regeneration through regulating DNA replication and cell division. We observed that the group of mice treated with an Hh inhibitor showed slow cell proliferation and arrest of mitosis, which caused the inhibition of liver regeneration. On the other hand, liver regeneration was inhibited in mice treated with the Hh inhibitor vismodegib, which was accompanied by a conspicuously reduced expression of Hh-inducible factors GLI1 and GLI2. Furthermore, we demonstrated that the decline in liver regeneration was due to interference of the cell cycle. In the G1/S phase, inhibition of Cyclin D and CDK2 in the vismodegib-treated group resulted in the suppression of DNA replication, which resulted in a reduction in cell proliferation. Recent studies have demonstrated Cyclin D as the downstream effector of the Hh signaling pathway [26]. In this study, we also identified Cyclin B as an effector molecule of Hh signaling. Our results show that accumulation of Cyclin B was observed in the vismodegib-treated group, which impeded liver cells from exiting mitosis while giving rise to a higher proportion of polyploid hepatocytes.

However, there are still many unresolved issues between Hh signaling and liver regeneration. There is some controversy over Hh pathway activation in the healthy liver of mammals. Most studies have shown that there is almost no detectable Hh signaling pathway in a healthy liver. However, research on conditional knock-out SMO in mice hepatocytes seemed to prove the existence of the Hh signaling pathway in a mature healthy liver [48,49,50]. Furthermore, studies conducted on a nonalcoholic fatty liver mouse model revealed that the Hh signal in hepatocytes could regulate hepatic inflammation [51,52,53]. Moreover, liver regeneration following PH induction was mediated in a different fashion from normal homeostasis taking place in a healthy liver. Our result also confirms the hypothesis that the Hh ligand IHH is detected only in non-parenchymal cells and enhanced after PH. However, the receptor SMO and intracellular signaling molecules GLI1 and GLI2 were detected in hepatocytes 2 days after PH, which is similar to a previous finding on regeneration after the novel two-staged hepatectomy (ALPPS surgery) on mice [47]. These results show the activation of the Hh signaling pathway in hepatocytes in liver regeneration after PH.

In conclusion, we demonstrated the important role of the Hh signal in liver regeneration, and we speculate that non-parenchymal cell-derived Hh signals act on hepatocytes and promote hepatocyte proliferation during liver regeneration after PH. Regulating Hh signaling may contribute to the recovery of liver mass after liver resection in clinical practice.

## Data Availability

Not applicable.

## Supplementary Materials

The following supporting information can be downloaded at: https://www.mdpi.com/article/10.3390/cimb44020032/s1, Table S1: Primer sequence of genes.

## Abbreviations

Hh	hedgehog
ICR	Institute of Cancer Research
PH	partial hepatectomy
PFA	paraformaldehyde
HGF	hepatocyte growth factor
EGF	epidermal growth factor
TGFβ	transforming growth factor-β
HSC	hepatic stellate cell
PTC	patched
SMO	smoothened
MPF	maturation-promoting factor
GLI	glioma-associated oncogene
FDA	Food and Drug Administration
RIPA	Radio Immunoprecipitation Assay
SDS-PEGA	sodium dodecyl sulfate polyacrylamide gel electrophoresis
PVDF	polyvinylidene fluoride
Edu	5-Ethynyl-2′-deoxyuridine
IHH	Indian hedgehog
APC/C	anaphase-promoting complex/cyclosome
CMC-NA	sodium carboxymethylcellulose
