# Supplementary material for: Inhibition of Hedgehog Delays Liver Regeneration through Disrupting the Cell Cycle
Source: Curr Issues Mol Biol. 2022 Jan 18;44(2):470–82. doi: 10.3390/cimb44020032 (PMC8928988; doi:10.3390/cimb44020032)
Supplement: Supplementary file 1 [file cimb-44-00032-s001.zip › cimb-1507312-supplementary.pdf]

current issues in molecular biology

MDPI

Article

Inhibition of Hedgehog Delays Liver Regeneration through Disrupting the Cell Cycle

Table S1. Primer sequence of genes.

Name		Sequence
Gli1	forward	GGAATTCGTGTGCCATTGGG
	reverse	AGGCGTGAATAGGACTTCCG
Gli2	forward	GATGCCAACCAGAACAAGCAG
	reverse	AGTCATCCCTGTCCAGGTCTT
Gli3	forward	GAAGAAACGCAATCACTATGCAG
	reverse	GTCCCACGGTAAGGGAGAGA
Ptc	forward	GCATTCTGGCCCTAGCAATA
	reverse	GGTAGCTCTCATAGCCTGGA
Ihh	forward	GAACTCACTGGCCATCTCTGT
	reverse	TATTTCGGTCACGGTCTGAGG
Cyclin D1	forward	TGACTGCCGAGAAGTTGTGC
	reverse	CTCATCCGCCTCTGGCATT
Cyclin B1	forward	AAGGTGCCTGTGTGTGAACC
	reverse	GTCAGCCCCATCATCTGCG
CDK1	forward	AGGTACTTACGGTGTGGTGTAT
	reverse	CTCGCTTTCAAGTCTGATCTTCT
CDK2	forward	ATGGAGAACTTCCAAAAGGTGG
	reverse	CAGTCTCAGTGTCGAGCCG
APC2	forward	GCCTGGAATACTGTGAGCACC
	reverse	ATTGCCTTGAAGGTCGTTCTG
APC11	forward	GATGGCGTTTAATGGCTGCTG
	reverse	TCCACTCCTGGCGACACAT
CDC20	forward	GTTCGTGTTCGAGAGCGATTT
	reverse	CTAGGGGTGGTCTGAACCTT
Bub1b	forward	AACAGAACTACCCTCAAGGGG
	reverse	GGCAAGGGAAACGCCAATTC
